# Abuse in Colombian elderly and its association with socioeconomic conditions and functionality

**DOI:** 10.25100/cm.v50i2.4013

**Published:** 2019-06-30

**Authors:** Carmen-Lucia Curcio, Claudia Payán-Villamizar, Abelardo Jiménez, Fernando Gómez

**Affiliations:** 1 Universidad de Caldas, Grupo de Investigaciones en Gerontología y Geriatría, Manizales, Colombia.; 2 Universidad del Valle, Grupo Gerontología y Geriatría, Cali, Colombia; 3 Fundación Universitaria San Martín, Medicina, Public Health Research Group GISAP, Cali, Colombia; 4 Universidad del Valle, Escuela de Salud Pública, Cali, Colombia

**Keywords:** Abuse, elderly, aged, aging, family, elder abuse, physical abuse, domestic violence, surveys and questionnaires, caregivers, socioeconomic factors, social class, Colombia, maltrato, adulto mayor, envejecimiento, familia, abuso al anciano, abuso fisico, violencia domestica, encuestas y cuestionarios, cuidadores, factores socioeconomicos, clase social, Colombia

## Abstract

**Objective:**

To describe the presence of abuse in elderly people in Colombia and its association with socio-demographic and functional conditions.

**Methods:**

Cross-sectional and descriptive research. Data were taken from the SABE Colombia Survey, a population study, with a national representative sample of 23,694 adults aged over 60 years. Presence and type of abuse by partners or family members, members were investigated. Generalized linear models with Poisson link function were used to estimate the causes of the prevalence of abuse by area of residence, region, age, sex, dependence on activities of daily living and living arrangements.

**Results:**

15.1% of the elderly in Colombia reported some type of abuse, and over 50% reported more than one form of abuse. Abuse proportion is greater in people who are aged 60-69, in women, people with lower levels of education, people who belong to lower socioeconomic status, people who live alone, people who live with children, and people in urban areas. The most frequent abuse form is psychological, followed by neglect and physical abuse. Dependence on basic and instrumental daily living activities increases the probabilities of suffering abuse.

**Conclusions:**

Home is a risky place for the elderly people, especially for those with functional dependence, those who belong to low socioeconomic strata and women. Results should encourage debate among researchers, professionals and decision makers on public policy about necessary actions and means to change violent family dynamics in homes with elderly people.

## Resumen

**Objetivo:**

Describir la presencia de maltrato en las personas mayores en Colombia y su asociación con condiciones sociodemográficas y funcionales.

**Métodos:**

Estudio transversal y descriptivo. Se tomaron los datos de la Encuesta SABE Colombia, un estudio poblacional, con una muestra representativa a nivel nacional de 23,694 adultos mayores de 60 años. Se indagó por la presencia y tipo de maltrato, por parte de los miembros de la familia, hogar o convivientes. Se utilizaron modelos lineares generalizados con función de vínculo de Poisson para estimar las razones de prevalencia de maltrato por zona de residencia, región, edad, sexo, dependencia en actividades de la vida diaria y convivencia.

**Resultados:**

Reportaron algún tipo de maltrato el 15.1% de las personas mayores en Colombia y más de la mitad refiere más de una forma de maltrato. Hay mayor proporción de maltrato en las personas de 60-69 años, en mujeres, con menor escolaridad, quienes viven en estratos bajos, solos, con hijos y en zona urbana. El maltrato más frecuente es el psicológico, seguido de negligencia y maltrato físico. La dependencia en actividades básicas e instrumentales de la vida diaria, incrementa la probabilidad de sufrir maltrato.

**Conclusiones:**

El hogar es un lugar de riesgo para los adultos mayores, especialmente aquellos con dependencia funcional, de estratos bajos y mujeres. Los resultados deberán animar el debate entre investigadores, profesionales y tomadores de decisiones de política publica, en torno a las acciones y los medios necesarios para transformar las dinámicas familiares violentas en hogares con personas mayores.

**Remark**

**Table:** 

**1)Why was this study done?**
The study of abuse is a global public health priority, which has been neglected, especially in comparison with other types of violence. Since older adults abuse is a social problem, it also has general long-term effects on society. However, these effects are poorly understood, not well documented, and not always easily detected. Elderly abuse poses a challenge to the region, given the accelerated ageing of its population, the strong influence of the family as a social support in old age, and the negative impact on health. In Colombia, the frequency, types and factors associated with elder abuse in a national sample are little known.
**2) What did the researchers do and find?**
In a representative sample of older adults in Colombia, the prevalence of abuse in this study is 15.1%; and over 50% report more than one type of abuse. The most frequent type of abuse was psychological, followed by neglect, physical, economic and sexual abuse. Being a woman, the dependence on daily living activities, living in urban areas and belonging to the lower socioeconomic strata increases the likelihood of some form of abuse.
**3) What do these findings mean?**
The high rates of abuse in elderly suggest that increased attention to this problem is justified, including investment in development and assessment of elder abuse interventions, in order to help reduce the spread and effect of elder abuse. Preventing mistreatment of older adults involves actions aimed at reducing risk situations for older people, their family and friends, promoting positive attitudes toward older people, and raising awareness. In particular, it involves actions to inform and educate the general public about the risk factors for mistreatment, and about best practices for reducing the risk that such situations may arise.

## Introduction

Elder abuse is a global problem that affects the health and human rights of millions of elderly people, worldwide. It is mentioned that about 1 out of 6 elderly people experienced any form of abuse in the last year, therefore, it is a problem that requires attention ^1^ ^-^ ^3^, since it is associated with health consequences and enormous social and economic costs ^2^ ^,^ ^4^ ^,^ ^5^.

Abuse can be defined in different ways, and all of them have led to considerable debates and questions ^2^ ^,^ ^5^. The most accepted one defines it as a single or repeated act that causes harm or suffering to an elderly person, or the lack of appropriate actions to prevent it, which occurs in a relationship based on trust. It can take various forms, such as physical, psychological, emotional, sexual, financial and neglect, which can be intentional or not ^3^ ^,^ ^6^. According to this definition, it is not the intentional or unintentional nature of abuse (single or repeated act) or neglect (lack of appropriate action) what matters, but its results, the presence of a trust relationship and the severity of the consequences, which may be evident or not. It should be noted that this definition excludes all forms of physical, moral or material abuse by strangers ^2^ ^,^ ^6^.

Comparing the prevalence of elder abuse in diverse studies is challenging, due to differences in the problem’s definition, the methodology used, the measuring instruments, the surroundings and the study population ^1^ ^,^ ^5^. It is estimated that the overall prevalence in communities in North and South America ranges between 2.2% and 44.6% ^5^. A comprehensive systematic review and a meta-analysis of the prevalence of abuse in the last year, in elderly adults within the community, of 38,544 identified articles, allowed to select 52 studies, carried out in 28 countries in diverse regions, including 12 countries that have low and medium incomes ^7^, using the already-mentioned WHO’s definition; it was estimated that 15.7% of people over 60 years old reported some form of abuse. The most frequent types were: psychological 11.6%, economic 6.8%, neglect 4.2%, physical 2.6% and sexual 0.9%. It is estimated that 1 out of 10 older adults is a victim of some form of abuse ^5^. According to the United Nations ^8^, the figures may be underestimated, since only one out of 24 cases is notified, partly because victims are usually afraid to report their relatives and friends. Around the world, the number of cases of elder abuse is expected to increase, given the rapid aging of population, and it will reach 320 million victims by 2050 ^7^ ^,^ ^8^.

In Latin America, it has been documented that prevalence varies between 5% and 86%, depending on the evaluated population and the methodology used, and it is higher in women ^9^ ^-^ ^13^.

In Colombia, government institutions, such as the National Institute of Legal Medicine, place elder abuse within domestic violence, and estimate an average population rate of 37.8 cases per 100,000 inhabitants, with a sustained increase since 2013, which is higher in women; and they refer to homes as the riskiest places for abuse on this population group. In this context, 82.4% of the aggressions occurred during the last year, mostly due to their relatives, children and siblings ^14^; these data are consistent with those of other countries in the region ^15^.

The following are identified as individual risk factors for abuse: being a woman, age, the degree of dependence of the victim, and the family relationship between the aggressor and the victim, among others ^2^ ^,^ ^5^ ^,^ ^7^. In addition, dependence on daily living activities (ADLs) is associated with increased risk of multiple forms of abuse ^16^.

Study of abuse is a global public health priority, which has been neglected, especially when compared to other types of violence ^7^. Elder abuse poses a challenge for the region, given the accelerated aging of its population ^17^, the great influence of the family as social support in elderly ^18^, and the negative consequences on health ^19^. In Colombia, the frequency, types and factors associated with elder abuse, in a national sample, are poorly understood. Therefore, this study aims to: describe the presence of domestic abuse on elder people in Colombia; and to examine its association with socio-demographic and functional conditions. Results should encourage debate among researchers, professionals from diverse disciplines, and decision makers on public policy, about the necessary actions and means to change violent family dynamics in homes with elderly people.

## Materials and Methods

### Design

Observational cross-sectional and descriptive study. Data were taken from the National Survey of Health, Welfare and Aging (SABE Colombia 2016), a cross-sectional population study with elderly adults living in urban and rural community areas of Colombia, all over the national territory ^20^ ^,^ ^21^.

### Population

SABE Colombia included a national representative sample of 23,694 adults aged over 60 years, living in Colombia. A multistage and stratified probabilistic sampling was made, with an estimate of the national representative single sample size and for each region ^20^

According to the population sizes, discriminated by sex, urban-rural location and five regions of the country, the expansion factors were calibrated in order to adjust the distribution differences observed between the planned sample and the collected sample ^22^. The overall response rate was 70% (62% for urban areas and 77% for rural areas) ^21^ ^,^ ^22^.

The main dependent variable was abuse. The presence and type of abuse within the last three months by family members, was investigated ^23^. Psychological abuse was evaluated with the following question: Have you experienced any of the following situations due to home members in the last three months? And the following answers were included as options: disrespectful or insulting behaviors, shouts or insults. Physical and sexual abuse were asked directly: have you experienced physical abuse or sexual assault in the last three months? In order to evaluate neglect, the presence of any of the following situations was included: medicine abuse, deprivation of social contacts, family abandonment, not being provided with extra resources when necessary, being sent to a nursing home and being left alone for days or weeks. Economic abuse was evaluated with a single question, which included the presence of any of the following situations: illegal or improper use or appropriation of property or personal finance; forced modification of the will or other legal documents; denial of the right to access and control personal funds; and fraud, blackmailing and scam. The answers were recorded on an ordinal scale and were dichotomized as “yes” and “no” for analysis. The number of abuse cases corresponds to the sum of all types of abuse. Finally, presence of abuse was defined by the relationship between elderly people who suffered some type of abuse and the total number of people included in this study ^23^.

### Covariates

The following covariates were included: sex, age group, socio-economic status, area of residence (rural/urban), country’s region and living arrangements. The socioeconomic area of residence was classified into six levels, and dichotomized as low for strata 1 and 2; and medium-high for the remaining strata. The country was divided into 6 regions: Atlantic, Central, Eastern, Pacific, Orinoquia-Amazonia and Bogotá. Age was categorized into three groups: 60-69 years old, 70-79, and over 80. Living arrangements was categorized according to whether the person lived alone, with their partner, with their children, with other relatives, or with friends; and they were analyzed separately. In order to evaluate functional independence in basic activities of daily living activities (BADLs), the Barthel index was used, which includes a set of basic functioning domains, such as urinary and fecal continence, motor skills, and the ability to carry out self-care activities on their own ^24^. People who obtained 100 points were considered independent. The IADLs refers to the person’s ability to perform the activities that are necessary to live independently in community, within their immediate surroundings; for their evaluation, it was used the modified Lawton and Brody scale ^25^, and the following activities were included: going shopping, cooking dinner, managing money, using phone, managing their medications, and using public transport; the persons capable of performing all activities without difficulty and without any help were qualified as independent.

### Ethical aspects

The ethics committees of the Universidad de Caldas and the Universidad del Valle approved this study, and participants gave their informed consent.

### Statistical analysis

Descriptive statistics with percentages for nominal and ordinal variables were used. The comparison of percentages was determined by the chi-squared test, in order to establish the differences between those who reported abuse and those who did not. Generalized linear models with Poisson link function and a robust correction of variance were used, in order to estimate the prevalence ratios (PR) of abuse. The presence of some type of abuse was the independent variable; and the analysis adjusted by area of residence, region, age, sex, dependence on ADL and IADL, and living arrangements were the dependent variables. Data were analyzed using SPSS version 25. A *p* <0.05 was considered statistically significant.

## Results

Overall, 15.1% of elderly people reported having suffered some type of abuse and over 50% reported more than one type of abuse. This percentage was higher in people aged 60-69 years (17.1%) (*p* <0.000); and in women (16.4% vs 13.6%) (*p* <0.000). There is a higher frequency of abuse in those with lower levels of education, (*p* <0.000) and those living in strata 1 and 2 (*p* <0.000). There is a higher percentage of abuse within the urban area, 79% of the total abuse (*p* <0.000). When analyzed by regions, there is a greater proportion of abuse in Orinoquia-Amazonia for all types of abuse, except for sexual abuse; however, it is important to note that the estimates of this region may be overestimated, given the coverage of sample collection in that area and its effect on the estimation error ^22^; as for the number of abuse cases, it is greater in the central region. There is a higher number of abuse cases in those living with their children, followed by those living with their grandchildren and with their partners (*p* <0.000) (Table 1).

**Table 1:** Distribution of abuse according to socio-demographic and functional characteristics of the elderly in Colombia.

Variables (%)	Psychological abuse N^**+**^ = 693,276	Physical abuse N^**+**^ = 105,832	Sexual abuse N^**+**^ = 12,961	Economic abuse N^**+**^ = 66,563	Neglect N^**+**^ = 449,850	Any form of abuse N^**+**^ = 801,388
**Sex**						
Men	11.7	1.5	0.1	1.3	8.3	13.6
Women	14.2	2.4	0.4	1.2	8.6	16.4
**Age**						
60-69	14.9	2.2	0.3	1.4	9.2	17.1
70-79	12.7	2.1	0.2	1.3	8.9	15.0
80+	6.2	0.6	0.1	0.6	4.7	7.0
**School education**						
< 5 years	12.5	2.2	0.2	1.2	8.3	14.7
> 5 years	14.4	1.6	0.4	1.4	8.8	16.1
**Stratum**						
Low: 1 y 2	13.7	2.1	0.3	1.3	9.1	16.0
Middle: 3 a 6	11.8	1.7	0.1	1.1	7.3	13.4
**Zone**						
Urban	13.3	1.7	0.4	1.1	9.4	15.3
Rural	12.4	2.1	0.2	1.7	8.2	14.6
**Region**						
Atlantic	9.7	1.3	0.4	1.2	6.0	10.9
Central	11.6	3.0	0.2	1.2	6.4	13.8
Pacific	14.0	2.1	0.3	1.1	11.3	16.5
East	13.6	1.5	0.2	1.1	8.8	15.6
Orinoquia-Amazonia^++^	19.8	10.5	0.1	9.3	20.7	32.3
Bogotá	17.2	1.2	0.1	1.4	10.9	19.0
**Living arrangements**						
Alone	12.3	2.1	0.1	3.7	18.2	23.0
Partner	13.2	2.1	0.3	1.6	9.6	15.3
Children	13.9	2.0	0.4	1.7	10.3	16.0
Grandchildren	13.8	2.0	0.2	1.3	9.0	15.7
Relatives/siblings	13.1	1.7	0.2	1.3	8.5	15.2
**Functionality**						
Dependence on ADL*	14.8	2.9	0.3	1.7	10.5	16.7
Dependence on IADL**	11.0	2.1	0.6	1.3	8.3	13.4
Total	13.1	2.0	0.2	1.3	8.5	15.1

+ Weighted sample size

++ Estimation error for this region is greater than the designed for sampling

* ADL: Basic activities of daily living

**IADL: Instrumental activities of daily living

Specifically, it was found that psychological is the most frequent form of abuse (13.1%), followed by neglect (8.5%), physical abuse (2.0%), economic abuse (1.3%), and sexual abuse (0.2%). All types of abuse were more frequent in women than in men, with significant differences (*p* <0.000). According to socioeconomic strata, there is an evident gradient: the higher the stratum, the lower the abuse frequency (*p* <0.000); all types of abuse are more frequent in people who belong to a low stratum. Abuse was reported to be more frequent in the rural area than in the urban area, with significant differences (*p* <0.000). Table 1 shows the distribution of the type of abuse according to socio-demographic characteristics.

For living arrangements, 9.1% of the elderly live alone, 49.6% with their partners, 59.7% with their children, 37.5% with their grandchildren, 17.4% with their relatives and/or siblings, and 2.0% with friends. The overall average of cohabiting members was 3.1. As for those who report abuse, 13.9% of them live alone, almost 50% live with their partners, over 50% live with their children, a third live with their grandchildren, less than a quarter live with their siblings or other relatives, and a small proportion live with their friends, with significant differences between all cohabiting members. The greater the number of cohabiting members, the lower the abuse frequency (*p* <0.000). For almost all types of abuse, there is a greater abuse frequency in people living with their children (*p* <0.000), except for sexual abuse, where the highest frequency corresponds to those living with their grandchildren (45.9%). Those who live with their partners also reported a high prevalence of abuse, ranging from 35.4% to 49.2%. About a fifth of people who live alone also reported abuse, with lower percentages of physical abuse (9.8%) and greater of economic abuse (26.6%).

Regarding functionality, 78.8% of the elderly are independent in ADL and 61.6% in IADL. From people who reported some type of abuse, 23.3% are dependent in ADL, and 34.0% in IADL, both with significant statistical differences (*p* <0.000). The percentages of abuse are higher in those who depend in IADL, except for sexual abuse, which is higher in those who depend in ADL (49.3%).

Although all the variables show significant differences between those who report abuse and those who do not, Poisson regression analysis shows that being a woman, dependence in ADL and IADL, living in urban areas, and belonging to strata 1 and 2 increases the likelihood of suffering some types of abuse (*p* <0.000). The prevalence ratios are shown in Table 2.

**Table 2:** Prevalence ratios of abuse in older adults, adjusted by covariates.

Variable	Number of abuse cases	
	PRs	95% CI
**Sex**		
Women	1.13	1.13-1.14
**Region**		
Bogotá	0.69	0.68- 0.70
Pacific	0.59	0.58-0.60
East	0.51	0.51-0.52
Central	0.46	0.45-0.46
Atlantic	0.37	0.36-0.37
Orinoquia-Amazonia	Ref	
**Zone**		
Urban	1.06	1.05-1.06
Stratum		
Low (1 and 2)	1.30	1.29-1.30
**Living arrangements**		
Alone	0.47	0.47- 0.47
Partner	0.97	0.96 - 0.97
Children	0.97	0.97-0.98
Grandchildren	0.94	0.94 - 0.95
Relatives/siblings	0.82	0.81-0.82
**Functionality**		
Dependence on ADL*	1.53	1.53-1.54
Dependence on IADL**	1.31	1.30-1.32

PRs, estimated prevalence ratios; models adjusted by region, area and dependence on ADL and ADL, living arrangements: partner, children, grandchildren, relatives/siblings and friends.

* ADL: Basic activities of daily living

**IADL: Instrumental activities of daily living

## Discussion

This study reveals that home is a risky place for elderly adults, especially those with functional dependence, those who live in urban areas, those who belong to low strata, and women.

The prevalence of abuse in this study (15.1%) is similar to that reported by Yon et al. ^7^, who, by means of a meta-analysis that included 52 studies published between 2002 and 2015, and which used the same definition of this study, estimated the global prevalence of abuse on elderly people by 15.7%; and although they mention considerable regional variations, specifically in the region of the Americas, the estimated prevalence does not differ from the global one.

The most frequent type of abuse was psychological and the least frequent was sexual, which are similar findings to those reported in the literature ^5^ ^,^ ^7^. Yon *et al*. ^7^, show similar prevalences to those found in Colombia; according to them, among the most frequent types of abuse, there can be found: psychological 11.6%, economic 6.8%, neglect 4.2%, physical 2.6% and sexual 0.9%. These figures slightly differ from those found in Colombian elderly adults, in whom a lower frequency of sexual and economic abuse was found, as well as an equal frequency of physical abuse, a similar frequency of psychological abuse and a greater frequency of neglect. When compared with data from the United States ^26^, it is evident that prevalence of psychological abuse and neglect are higher in Colombia. As mentioned in the literature, differences in global prevalence and in different types of abuse may be due to report or sub-report problems, because of lack of knowledge, lack of experience, shame, being afraid of reprisals, being afraid to be wrong, being afraid of abuse being increased, or because of an emotional bond ^26^ ^-^ ^29^.

It has also been mentioned that the record and measurement forms are insufficient and underdeveloped in the region ^30^. In fact, Acierno *et al*., found that approximately 11% of elderly adults experience abuse each year, but only 1 out of 14 cases is reported to authorities; and it is suggested that for every documented abuse case, 24 cases are not reported ^27^ ^,^ ^31^. According to the *First inter-sectorial report, violence against the elderly people*, of the Mayor Office of Bogotá ^32^, some situations of abuse are evident, especially in cases related to physical aspects and neglect of the elderly person, which makes it easier for identification and intervention by society and the State; but other types of violence, such as sexual, psychic, emotional or economic abuse are more difficult to detect and are not reported, either because the elderly people have got used to this type of relationship, because of being afraid of losing their network support, or because of naturalization of this situation within families ^32^.

On the other hand, findings highlight the fact that dependence on basic and instrumental activities of daily living has the highest prevalence ratios in this study. Evidence shows the relation between abuse and dependence, especially within the family context ^16^ ^,^ ^33^. Several studies confirm the association between dependency for ADL and abuse ^16^ ^,^ ^29^ ^,^ ^34^. When dependency arises, elderly people need increasing support, which is in most cases provided exclusively by relatives, who are exposed to stress, tension and conflict situations, since generally there is only one person who provides the required support. In addition, since IADL refer to the independence of the elderly person within home and, especially in the community, under high complexity conditions ^35^, this may be one of the reasons why dependence on IADL is associated with abuse, since the elderly person has limitations for going out, using transport or staying into a support network, and looking for support, in order to stop the abuse situation ^13^. Dependence on IADL limits independent social participation of elderly people, restricting them to be inside their homes; it can also cut down their relationships with other people, besides their relatives, hindering their demand of health services and specialized services that can help them to report abuse, when subjected to it ^13^.

It has been suggested that disability is both a risk factor and a consequence of abuse; the risk is attributed to a set of complex contextual and social factors: greater dependence on others for care, physical vulnerability, social isolation and lack of economic independence make people with disabilities more vulnerable ^36^. The first theories on this subject also tried to establish whether there was a relation between dependence and a higher risk of suffering abuse ^37^. Initially, the relationship of dependence of the victim with the caregiver or the aggressor was emphasized, although subsequent study cases made possible to detect situations in which the aggressors depended on the elderly person; in general, adult children (sons and daughters) who depend on their elderly parents for accommodation or livelihood ^38^. In some of these cases, the existence of “entangled interdependencies” was evident; *i.e.*, a very strong emotional bond between the victim and the aggressor, which often hinders intervention ^37^. It has also been proposed that dependence of the elderly people may force the caregiver to bring up past hostilities, resulting in abuse of the elderly people ^31^.

Regarding the socioeconomic status, the report of abuse in this study is considerably high in strata 1 and 2 (71.7%), and it is also a predictive factor (PRs: 1.30); however, there are few studies that refer to it. A study from The United Kingdom ^39^, although not considering the stratum but the socioeconomic position, found higher rates among those who had been in semi-routine and routine occupations (4.1%), compared to those who had been small employers and self-employed (0.1 %); and although the figures are not comparable, they are much lower than those found in this study. On the other hand, regarding the level of education in Colombia, an inversely proportional association was found between the level of education and violence against the elderly population ^14^.

The World Report on Violence and Health ^37^ sets a link between poverty and violence, and describes how a dysfunctional family life, the lack of money for essential items, and the lack of education and job opportunities contribute to young people dedicating themselves to crime, drug trafficking and prostitution. In this type of societies, elderly people are considered a target for abuse and exploitation, because their vulnerability is the result of a poverty situation, characterized by the lack of a retirement and job opportunities, lack of hygiene, disease and malnutrition.

Regarding sex, abuse in women is more frequent than in men, among elderly adults ^19^ ^,^ ^20^. Throughout the world, men are more likely than women to suffer violence in the context of armed conflicts and criminal activities, while women are more likely to suffer abuse from close people, such as their partners and family ^37^. This gender asymmetry in domestic abuse seems to occur as a result of the expression of a violence background, or as a response to a conflict that has been present in the relationship throughout life, but that worsens when something happens altering the balance, as it happens with retirement ^40^. Elderly women may have more barriers for looking for support, and may be less aware of available support ^1^. It is important to consider that current elderly women grew up in societies with sharp gender roles, and they learned to be subordinated ^41^. Thus, elderly women tolerate more and are less prepared to cut off abusive relationships ^40^. Acceptability of violence against women inflicted by their intimate partner is widespread, but it varies according to the surroundings; moreover, the agreement with norms that strengthen gender inequalities, discourage women from asking for help, or downplay third parties’ responsibility to intervene in abuse situations is widespread in the region ^40^.

According to data of the Legal Medicine Office, during 2018 in Colombia, for all categories of domestic violence, women were overall the most victimized; in addition, people aged between 60 and 64 years are the most affected; the risk is higher if the person has only primary elementary education and if is married; it is important to point out that for this entity, the term intra-family violence refers to any form of abuse, whether physical, psychological or sexual, that takes place among family members; as happens with every form of abuse, it implies a power imbalance, and it is exercised from the strongest to the weakest person, in order to exercise control over the relationship. Traditionally, in our society, within the currently predominant hierarchical family structure, the two main axes of imbalance have been composed by gender and age, with women, children and the elderly people being the main victims of violence within the family ^42^.

In addition, current evidence supports the existence of new arrangements within families, and a growing and complex family diversity; although the nuclear family type (a couple with children) is still predominant, there are also unusual forms of family organization; for example: grandparents (as a couple or alone) living with their grandchildren, grandparents with grandchildren and great-grandchildren; and simple single-parent, composed and extensive homes, among others. The reality faced by families regarding to care of the elderly people is complicated; changes generated inside, in addition to the family-centered system, overwhelm families, since they are the ones who assume care responsibilities. Similarly, the absence of public policies that support this work makes dependent elderly adults even more vulnerable to the likelihood of abuse, both within family and within institutions that provide care ^43^. In this context, the family constitutes the main providing support pillar; and somehow, it covers the social risks derived from the aging process; however, there have been transformations within family, in this sense, as there is no solid State support that contributes to improving life conditions of the elderly people and their families; the latter is subject to an overload in economic, social and care terms, which in many cases result in abuse.

In this family context, it has been found that direct family members are usually the main abuse performers against elderly adults, especially children and partners ^42^. It has also been suggested that abuse comes from the caregiver’s dependence on the elderly person, either financially, for transport or for accommodation, and not from the victim’s dependence on the abuser ^44^ ^,^ ^45^. Several authors have suggested a change of approach from the victim to the abuser ^44^ ^-^ ^46^, and that the interdependence of roles could explain the aggression towards the elders ^47^; thus, abuse can be a manifestation of the dependence of the abusers and not of the elderly, the most likely cause being a power imbalance involving a “perceived power deficit” of an *adult-son* ^48^. Abusive family members may depend on the elderly for accommodation, transport and financial support, and the perceived power imbalance can be especially tough for adult children or grandchildren who live with the elderly person; family members can resort to aggression, in order to try to get a power advantage and overcome their perceived power deficit.

On the other hand, many elderly people do not leave abusive situations, and often deny abuse is occurring, arguing that they are also getting something from the exchange relationship ^44^. The Family Care Dynamics Model argues that role interdependence is a partial explanation of this phenomenon ^49^. In families, both members and elders depend on each other for love, respect, companionship and emotional support. This interdependence of roles creates a cycle of negative reinforcement, which does not allow any of the members to cut off the relationship, and abuse keeps on ^48^ ^,^ ^49^. In addition, according to the reports of the Institute of Legal Medicine ^14^ the concept of inter-generational transmission of violence has been used to explain how violence is learned in the context of family socialization; that is, the observation of violence in the family context can influence children to learn to exercise violence against other people ^50^. A significant association between current abuse and family background has been demonstrated, resulting in a phenomenon of inter-generational reproduction of violence ^51^.

Finally, this study found a higher percentage of abused people within the younger age group, from 60 to 70 years old, as it has also been reported in Colombia ^14^, and in other studies ^1^. It has been proposed that this age group of younger elders is the one where the main complaints of abuse arise ^4^ ^,^ ^19^. In that age group, many of the elderly people are physically and intellectually active and have more autonomy, independence and conditions to look for support. In contrast, the scientific literature reports that the greatest victims are the oldest people, due to functional and cognitive limitations ^5^ ^,^ ^31^.

One of the greatest strengths of this study is its population nature, which makes it possible to generalize the results throughout the country. In our knowledge, this is the first Colombian study that shows the prevalence of abuse in a general and specific way by types of abuse, and that analyzes its relations with the region, the geographical area, the socioeconomic stratum, family arrangements and functional dependence in elderly adults.

Among the limitations of this study, there can be found that data were recorded by self-report, with a scale that do not measure neither the intentionality nor the severity of abuse, which prevents estimating more accurately the differences in experience and perpetuation. In addition, the specific source of abuse was not precisely identified. Finally, this is a cross-sectional and quantitative study on abuse. Future studies on this subject should include a qualitative approach, as well as methodologies that can establish causal relations.

## Conclusions

Elder abuse should be a priority for public and health policy schedules over the country. This study exposes that home is a risky place for elderly adults, especially those with functional dependence, those who belong to low strata, and women. From a social perspective, the most serious consequence of violence will be isolation, decreased self-esteem, and the presence of feelings of distrust, which will stimulate creation of negative stereotypes towards elderly. From a public health perspective, elder abuse can lead to serious consequences; the isolated not-supported elderly adult is more vulnerable and will suffer a greater morbidity and disability. These results should encourage debate on the relationship patterns within Latin American families, and the role of women within them, and lead to think of the necessary means to change violent family dynamics within homes with elderly people. 

## References

[1] Pathak N, Dhairyawan R, Tariq S (2019). The experience of intimate partner violence among older women A narrative review. Maturitas.

[2] Beaulieu M, Leboeuf R, Pelletier C, Laforest J, Maurice P, Bouchard LM (2019). Mistreatment of older adults. Québec Report on Violence and Health.

[3] World Health Organization (2018). Elder Abuse - Fact Sheet 357.

[4] European Institute for Gender Equality (2014). Estimating the costs of gender-based violence in the European Union Report.

[5] Dong XQ (2015). Elder abuse Systematic review and implications for practice. J Am Geriatr Soc.

[6] Organización Mundial de la Salud (2002). Declaración de Toronto para la prevención global del maltrato a las personas mayores. Rev Española Geriatr Gerontol.

[7] Yon Y, Mikton CR, Gassoumis ZD, Wilber KH (2017). Elder abuse prevalence in community settings a systematic review and meta-analysis. Lancet Glob Health,.

[8] United Nations (2018). World Elder Abuse Awareness Day 15 June.

[9] Piña-Escudero SD, Weinstein CA, Ritchie C (2019). Contextualizing mistreatment in cognitive impairment in Latin America. J Elder Abuse Negl.

[10] Guedes DT, Curcio CL, Llano BA, Zunzunegui MV, Guerra R (2015). The gender gap in domestic violence in older adults in Latin America The IMIAS Study. Rev Panam Salud Publica.

[11] Guedes DT, Alvarado BE, Phillips SP, Curcio CL, Zunzunegui MV, Guerra RO (2015). Socioeconomic status, social relations and domestic violence (DV) against elderly people in Canada, Albania, Colombia and Brazil. Arch Gerontol Geriatr.

[12] Guedes DT, Vafaei A, Alvarado BE, Curcio CL, Guralnik JM, Zunzunegui MV (2016). Experiences of violence across life course and its effects on mobility among participants in the International Mobility in Aging Study. BMJ Open.

[13] de Paiva MM, Tavares DM dos S (2015). Violência física e psicológica contra idosos prevalência e fatores associados. Rev Bras Enferm.

[14] Instituto Nacional de Medicina Legal y Ciencias Forenses (2018). Forensis 2018. Datos para la vida.

[15] Garmendia LF (2011). La violencia en América Latina. An Fac Med.

[16] Dong X, Simon M, Evans D (2014). A population-based study of physical function and risk for elder abuse reported to social service agency Findings from the Chicago health and aging project. J Appl Gerontol.

[17] Fríes L, Hurtado V (2010). Estudio de la información sobre la violencia contra la mujer en America Latina y el Caribe.

[18] Cardona-Arango D, Estrada-Restrepo A, Chavarriaga-Maya LM, Segura-Cardona ÁM, Ordoñez-Molina J, Osorio-Gómez JJ (2010). Apoyo social dignificante del adulto mayor institucionalizado Medellín, 2008. Rev Salud Pública.

[19] Sherin KM, Sinacore JM, Li XQ, Zitter RE, Shakil A (1998). HITS a short domestic violence screening tool for use in a family practice setting. Fam Med.

[20] Gomez F, Corchuelo J, Curcio CL, Calzada MT, Mendez F (2016). SABE Colombia Survey on Health, Well-Being, and Aging in Colombia - Study Design and Protocol. Curr Gerontol Geriatr Res.

[21] Ministerio de Salud y Protección Social, COLCIENCIAS, Universidad del Valle, Universidad de Caldas (2016). Encuesta SABE Colombia: Situación de Salud, Bienestar y Envejecimiento en Colombia.

[22] Ministerio de Salud y Protección Social (2018). Documento Metodológico Encuesta Nacional de Salud, Bienestar y Envejecimiento SABE Colombia.

[23] Huenchuan S (2008). Directrices para la elaboración de módulos sobre envejecimiento en las encuestas de hogares.

[24] Barthel D, Mahoney F (1965). Functional evaluation the Barthel Index. Md State Med J.

[25] Lawton MP, Brody EM (1969). Assessment of older people Self-maintaining and instrumental activities of daily living. Gerontologist.

[26] Sidani MA, Reed BC, Steinbauer J (2017). Geriatric Care Issues An American and an International Perspective. Primary Care.

[27] Acierno R, Hernandez-Tejada MA, Anetzberger GJ, Loew D, Muzzy W (2017). The national elder mistreatment study An eight-year longitudinal study of outcomes. J Elder Abuse Negl.

[28] Jackson SL (2016). All elder abuse perpetrators are not alike the heterogeneity of elder abuse perpetrators and implications for intervention. Int J Offender Ther Comp Criminol.

[29] Johannesen M, LoGiudice D (2013). Elder abuse A systematic review of risk factors in community-dwelling elders. Age Ageing.

[30] Comisión Económica para América latina y el Caribe (2012). Los bonos en la mira. Aporte y carga para las mujeres..

[31] Acierno R, Hernandez MA, Amstadter AB, Resnick HS, Steve K, Muzzy W (2010). Prevalence and correlates of emotional, physical, sexual, and financial abuse and potential neglect in the United States The national elder mistreatment study. Am J Public Health.

[32] Alcaldía Mayor de Bogotá (2012). Violencias contra las personas mayores en el marco de las relaciones familiares.

[33] Iborra MI (2008). Factores de riesgo del maltrato de personas mayores en la familia en población española. Zerb Serv Soc.

[34] Pérez-Cárceles MD, Rubio L, Pereniguez JE, Pérez-Flores D, Osuna E, Luna A (2009). Suspicion of elder abuse in South Eastern Spain The extent and risk factors. Arch Gerontol Geriatr.

[35] Gomez F, Curcio C-L (2014). Salud del anciano: valoracion. Tercera.

[36] Breckenridge JP, Lombard N (2018). The relationship between disability and domestic abuse. The Routledge Handbook of Gender and Violence.

[37] Krug EG, Dahlberg LL, Mercy JA, Zwi AB, Lozano R (2003). Informe mundial sobre la violencia y la salud.

[38] Pillemer KA, Pillemer KA, Wolf RS (1986). Risk factors in elder abuse: Results from a case-control study. Elder abuse: Conflict in the family.

[39] Biggs S, Manthorpe J, Tinker A, Doyle M, Erens B (2009). Mistreatment of older people in the United Kingdom findings from the first national prevalence study. J Elder Abuse Negl.

[40] Bott S, Guedes A, Goodwin M, Mendoza JA (2012). Violence against women in Latin America and the Caribbean: A comparative analysis of population-based data from 12 countries.

[41] Farmer A, Tiefenthaler J (1997). An economic analysis of domestic violence. Rev Soc Econ.

[42] Arriagada I (2007). Familias latinoamericanas cambiantes, diversas y desiguales. Papeles Poblac.

[43] Pillemer K (1985). The dangers of dependency New findings on domestic violence against the elderly. Soc Probl.

[44] Pillemer K, Finkelhor D (1988). The prevalence of elder abuse a random sample survey. Gerontologist.

[45] Burnes D, Pillemer KA (2016). Elder abuse global situation, risk factors, and prevention strategies. Gerontologist.

[46] Goergen T, Beaulieu M (2013). Critical concepts in elder abuse research. Int Psychogeriatr.

[47] Pickering CE, Phillips LR (2014). Development of a causal model for elder mistreatment. Public Health Nurs.

[48] Phillips LR, Rempusheski VF (1986). Caring for the frail elderly at home toward a theoretical explanation of the dynamics of poor quality family caregiving. ANS Adv Nurs Sci.

[49] Gámez-Guadix M, Calvete E (2012). Violencia filioparental y su asociación con la exposición a la violencia marital y la agresión de padres a hijos. Psicothema.

[50] Flood M, Pease B (2009). Factors influencing attitudes to violence against women. Trauma, Violence Abuse.

[51] Duque AM, Leal MC, Marques AP, Eskinazi FM, Duque AM (2012). Violência contra idosos no ambiente doméstico prevalência e fatores associados (Recife/PE). Cien Saude Colet.

