# Supplementary material for: Abuse in Colombian elderly and its association with socioeconomic conditions and functionality
Source: Colomb Med (Cali). 2019 Jun 30;50(2):77–88. doi: 10.25100/cm.v50i2.4013 (PMC6774576; doi:10.25100/cm.v50i2.4013)
Supplement: Translation

# Translation: Maltrato en adultos mayores colombianos y su asociación con condiciones socioeconómicas y funcionalidad

**Contribución del estudio**

**Table:** 

**1) Por que se realizó este estudio?**
El estudio del maltrato es una prioridad mundial de salud pública descuidada, especialmente en comparación con otros tipos de violencia. Dado que el maltrato de adultos mayores es un problema social, también tiene efectos generales a largo plazo en la sociedad. Sin embargo, estos efectos no se entienden bien, no están bien documentados y no siempre son fáciles de detectar. El maltrato a las personas mayores plantea un desafío para la región dado el envejecimiento acelerado de su población, la gran influencia de la familia como apoyo social en la vejez, y las consecuencias negativas en la salud. En Colombia, la frecuencia, los tipos y los factores asociados con el maltrato de adultos mayores en una muestra nacional son poco conocidos.
**2) Cuales fueron los resultados relevantes del estudio?**
En una muestra representativa de adultos mayores en Colombia, la prevalencia de maltrato en este estudio es 15.1% y más de la mitad reporta más de un tipo de maltrato. El tipo de maltrato más frecuente fue el psicológico, seguido de negligencia, maltrato físico, económico y sexual (0.2%). Ser mujer, la dependencia en actividades de la vida diaria, vivir en zona urbana y pertenecer a estratos bajos incrementa la probabilidad de sufrir algún tipo de maltrato.
**3) Que aportan estos resultados?**
Las elevadas tasas de maltrato sugieren que se justifica prestar más atención a este problema, incluida la inversión en el desarrollo y la evaluación de las intervenciones para ayudar a reducir la propagación y el efecto del maltrato de las personas de edad. Prevenir el maltrato de adultos mayores implica acciones destinadas a reducir las situaciones de riesgo para las personas mayores y sus familiares y amigos, promover actitudes positivas hacia las personas mayores y aumentar la concientización. En particular, implica acciones para informar y educar al público en general sobre los factores de riesgo de malos tratos y sobre las mejores prácticas para reducir el riesgo de que se presente.

## Introducción

El maltrato de las personas mayores es un problema mundial que afecta a la salud y los derechos humanos de millones de personas mayores en todo el mundo. Se menciona que alrededor de 1 de cada 6 personas mayores experimentaron alguna forma de abuso en el último año, por tanto, es un problema que requiere atención ^1^^-^^3^ dado que se asocia con consecuencias en la salud y enormes costos sociales y económicos ^2^^,^^4^^,^^5^.

El maltrato se puede definir de diferentes maneras y todas han provocado considerables debates y preguntas ^2^^,^^5^ la más aceptada la define como un acto único o repetido que causa daño o sufrimiento a una persona de edad, o la falta de medidas apropiadas para evitarlo, que se produce en una relación basada en la confianza. Puede adoptar diversas formas como el maltrato físico, psíquico, emocional o sexual, financiero y descuido o negligencia, la cual puede ser intencional o no ^3^^,^^6^. De acuerdo con esta definición, no es la naturaleza intencional o no intencional del maltrato (acto único o repetido) o la negligencia (falta de acción apropiada) lo que importa, sino sus resultados, la presencia de una relación de confianza y la severidad de las consecuencias, evidentes o no. Cabe señalar que esta definición excluye todas las formas de abuso físico, moral o material por parte de extraños ^2^^,^^6^.

Comparar la prevalencia del maltrato a personas mayores en diversos estudios es un desafío, debido a las diferencias en la definición del problema, la metodología utilizada, los instrumentos de medición, el entorno y la población de estudio ^1^^,^^5^. Se estima que la prevalencia general en la comunidad en América del norte y del sur oscila entre 2.2% y 44.6% ^5^. Una amplia revisión sistemática y metanálisis en torno a la prevalencia de maltrato en el último año, en adultos mayores de la comunidad, de 38544 artículos identificados, seleccionó 52 estudios llevados a cabo en 28 países de diversas regiones, incluidos 12 países de ingresos bajos y medianos ^7^, utilizando la definición de la OMS ya mencionada, estimó que 15.7% de las personas de 60 años o más reportaron alguna forma de maltrato. Los tipos más frecuentes fueron: psicológico 11.6%, económico 6.8%, negligencia 4.2%, maltrato físico 2.6% y sexual 0.9%. Se estima que 1 de cada 10 adultos mayores es víctima de alguna forma de maltrato ^5^. Según Naciones Unidas ^8^ probablemente la cifra esté subestimada, puesto que solo se notifica uno de cada 24 casos, en parte porque los afectados suelen tener miedo de denunciar a sus familiares y amigos. En todo el mundo, se prevé que el número de casos de maltrato de personas mayores aumente dado el rápido envejecimiento de la población y llegará a 320 millones de víctimas para 2050 ^7^^,^^8^. 

En Latinoamérica se ha documentado que la prevalencia varía entre el 5% y el 86% según la población evaluada y la metodología utilizada y es mayor en mujeres ^9^^-^^13^. 

En Colombia, instituciones gubernamentales como el Instituto Nacional de Medicina Legal, ubican el maltrato al anciano dentro de la violencia intrafamiliar y estiman una tasa media poblacional de 37.8 casos por cada 100,000 habitantes, con un incremento sostenido desde 2013, mayor en mujeres, y referencian la vivienda como el lugar de mayor riesgo para el maltrato de este grupo poblacional; en este escenario ocurrieron durante el último año 82.4% de las agresiones, las cuales provienen en su mayoría de familiares, hijos y hermanos ^14^, estos datos concuerdan con los de otros países de la región ^15^.

Se señalan como factores de riesgo individuales para el maltrato: ser mujer, la edad, el grado de dependencia de la víctima y la vinculación familiar del agresor con la víctima, entre otros ^2^^,^^5^^,^^7^. Además, la dependencia en actividades de la vida diaria (AVD) se asocia con incremento del riesgo de sufrir múltiples formas de maltrato ^16^.

El estudio del maltrato es una prioridad mundial de salud pública descuidada, especialmente en comparación con otros tipos de violencia ^7^. El maltrato a las personas mayores plantea un desafío para la región dado el envejecimiento acelerado de su población ^17^, la gran influencia de la familia como apoyo social en la vejez ^18^, y las consecuencias negativas en la salud ^19^. En Colombia, la frecuencia, los tipos y los factores asociados con el maltrato de adultos mayores en una muestra nacional son poco conocidos. Por ello, este estudio tiene como objetivos: describir la presencia de maltrato en el hogar en las personas mayores en Colombia y examinar la asociación con condiciones sociodemográficas y funcionales. Los resultados deberán animar el debate entre investigadores, profesionales de diversas disciplinas y tomadores de decisiones de política publica, en torno a las acciones y los medios necesarios para transformar las dinámicas familiares violentas en hogares con personas mayores.

## Materiales y Métodos

### Diseño

Estudio observacional de corte transversal y descriptivo. Se tomaron los datos de la Encuesta Nacional de Salud, Bienestar y Envejecimiento (SABE Colombia 2016), un estudio poblacional, transversal con adultos mayores que viven en la comunidad en áreas urbanas y rurales de Colombia en todo el territorio nacional ^20^^,^^21^. 

### Población

El estudio SABE Colombia incluyó una muestra representativa a nivel nacional de 23.694 adultos mayores de 60 años que viven en Colombia. Se hizo un muestreo probabilístico polietapico y estratificado, con un cálculo de tamaño de muestra único representativo nacional y para cada región. Acorde con los tamaños de población desagregados por sexo, ubicación urbano-rural y cinco regiones del país, los factores de expansión fueron calibrados con el propósito de ajustar las diferencias de distribución observadas entre la muestra planeada y la muestra finalmente recolectada ^22^. La tasa de respuesta general fue del 70% (62% para áreas urbanas y 77% para áreas rurales) ^21^^,^^22^. 

La principal variable dependiente fue el maltrato. Se indagó por la presencia y tipo de maltrato en los últimos tres meses, por parte de los miembros de la familia, hogar o convivientes ^23^. El maltrato psicológico fue evaluado con la siguiente pregunta: Ha experimentado alguna de las siguientes situaciones por parte de los miembros de su hogar en los últimos tres meses? y se incluyeron como opciones de respuesta: comportamientos irrespetuosos o insultantes, gritos o insultos. El maltrato físico y el sexual se preguntaron directamente: en los últimos tres meses ha experimentado maltrato físico o agresiones sexuales?. Para evaluar negligencia se incluyó la presencia de alguna de las siguientes situaciones: el uso indebido de medicamentos, privación de contactos sociales, abandono familiar, no proporcionar recursos extras cuando eran necesarios, ser llevado a un ancianato o asilo y si lo habían dejado solo por días o semanas. El abuso económico se evaluó con una sola pregunta que incluyó la presencia de cualquiera de las siguientes situaciones: uso ilegal o indebido, o apropiación de la propiedad o finanzas personales, modificación forzada del testamento u otros documentos jurídicos, negación del derecho de acceso y control sobre los fondos personales y fraude, chantaje y estafa. Las respuestas se registraron en una escala ordinal y para el análisis se dicotomizaron en si y no. El número de maltratos corresponde a la sumatoria de todos los tipos de maltrato. Finalmente, la presencia de maltrato de definió por la relación entre las personas mayores que sufrieron algún tipo de maltrato y el total de personas incluidas en el estudio ^23^. 

### Covariables

Se incluyó sexo, grupo de edad, estrato socioeconómico, zona de residencia (rural/urbana), región del país y el tipo de convivencia. El estrato socioeconómico del lugar de residencia se definió en seis niveles y se dicotomizó en bajo (estratos 1 y 2) y medio-alto los restantes. Las regiones se clasificaron en 6: Atlántica, Central, Oriental, Pacífica, Orinoquia-Amazonia y Bogotá. La edad se categorizó en tres grupos 60-69 años, 70-79 y 80 y más. La convivencia se categorizó de acuerdo a si la persona vivía sola, con la pareja, con los hijos, con otros familiares y con amigos y se analizaron separadamente. Para la valoración de independencia funcional en las actividades básicas de la vida diaria (ABVD) se utilizó el índice de Barthel que incluye una serie de dominios básicos de funcionamiento, como continencia urinaria y fecal, movilidad y la capacidad para llevar a cabo de forma independiente actividades de autocuidado ^24^ se consideraron independientes las personas que obtuvieron 100 puntos. Las AIVD se refieren a la capacidad de la persona para realizar las actividades necesarias para vivir de manera independiente en la comunidad en su entorno inmediato, para su valoración se utilizó la escala de Lawton y Brody modificada ^25^ y se incluyeron las siguientes actividades: hacer la compra, preparar la comida, manejar dinero, usar el teléfono, manejar sus medicamentos y uso de transporte público; se calificó como independiente a la persona capaz de realizar todas las actividades sin dificultad y sin ayuda de nadie. 

### Aspectos éticos

Los comités de ética de la Universidad de Caldas y de la Universidad del Valle aprobaron el estudio y se obtuvo el consentimiento informado de los participantes.

### Análisis estadísticos

Se utilizó estadística descriptiva con porcentajes para las variables nominales y ordinales. La comparación de porcentajes se determinó con la prueba chi cuadrado para establecer las diferencias entre quienes reportaron maltrato y quiénes no. Se utilizaron modelos lineares generalizados con función de vínculo de Poisson y corrección robusta de la varianza para estimar las razones de prevalencia (RP) de maltrato. La presencia de algún tipo de maltrato fue la variable independiente y el análisis ajustado por zona de residencia, región, edad, sexo, dependencia en ABVD y AIVD y convivientes. Los datos fueron analizados utilizando SPSS versión 25. Una *p* <0.05 fue considerada estadísticamente significativa.

## Resultados

En general, 15.1% de las personas mayores reportó haber sufrido algún tipo de maltrato y más de la mitad reporta más de un tipo de maltrato. Fue mayor en las personas de 60-69 años (17.1%) (*p* <0.000) y en las mujeres (16.4% vs 13.6%) (*p* <0.000). Hay mayor proporción de maltrato en quienes tienen menor nivel de escolaridad (*p* <0.000) y de estratos 1 y 2 (*p* <0.000). Se encontró una mayor proporción de maltrato en la zona urbana, 79% del total de maltratados (*p* <0.000). Por regiones, hay mayor proporción de maltrato en la Orinoquia - Amazonia en todos los tipos de maltrato, excepto el sexual, sin embargo, es importante anotar que las estimaciones de esta región pueden estar sobreestimadas dada la cobertura de recolección de muestra en dicha zona y su efecto en el error de estimación ^22^; en cuanto al número de maltratos, es mayor en la región central. Hay mayor proporción de maltrato en quienes viven con hijos, seguidos de quienes viven con los nietos y con el cónyuge (*p* <0.000). (Tabla 1). 

**Tabla 1:** Distribución del maltrato según características sociodemográficas y funcionales de las personas mayores en Colombia.

Variables (%)	Maltrato Psicológico N^**+**^ = 693,276	Maltrato Físico N^**+**^ = 105,832	Maltrato Sexual N^**+**^ = 12,961	Maltrato Económico N^**+**^ = 66,563	Negligencia N^**+**^ = 449,850	Con algún tipo de maltrato N^**+**^ = 801,388
**Sexo **						
Hombres	11.7	1.5	0.1	1.3	8.3	13.6
Mujeres	14.2	2.4	0.4	1.2	8.6	16.4
**Edad**						
60-69	14.9	2.2	0.3	1.4	9.2	17.1
70-79	12.7	2.1	0.2	1.3	8.9	15.0
80+	6.2	0.6	0.1	0.6	4.7	7.0
**Escolaridad**						
< 5 años	12.5	2.2	0.2	1.2	8.3	14.7
> 5 años	14.4	1.6	0.4	1.4	8.8	16.1
**Estrato**						
Bajo: 1 y 2	13.7	2.1	0.3	1.3	9.1	16.0
Medio: 3 a 6	11.8	1.7	0.1	1.1	7.3	13.4
**Zona**						
Urbana	13.3	1.7	0.4	1.1	9.4	15.3
Rural	12.4	2.1	0.2	1.7	8.2	14.6
**Region**						
Atlántica	9.7	1.3	0.4	1.2	6.0	10.9
Central	11.6	3.0	0.2	1.2	6.4	13.8
Pacifica	14.0	2.1	0.3	1.1	11.3	16.5
Oriental	13.6	1.5	0.2	1.1	8.8	15.6
Orinoquia-amazonia^++^	19.8	10.5	0.1	9.3	20.7	32.3
Bogotá	17.2	1.2	0.1	1.4	10.9	19.0
**Convivientes**						
Solo	12.3	2.1	0.1	3.7	18.2	23.0
Cónyuge	13.2	2.1	0.3	1.6	9.6	15.3
Hijos	13.9	2.0	0.4	1.7	10.3	16.0
Nietos	13.8	2.0	0.2	1.3	9.0	15.7
Parientes/hermanos	13.1	1.7	0.2	1.3	8.5	15.2
**Funcionalidad**						
Dependencia en ABVD*	14.8	2.9	0.3	1.7	10.5	16.7
Dependencia en AIVD**	11.0	2.1	0.6	1.3	8.3	13.4
Total	13.1	2.0	0.2	1.3	8.5	15.1

^+^ Tamaño de muestra ponderado

^++^ El error de estimación para esta región es mayor al diseñado para el muestreo

^*^ ABVD: Actividades básicas de la vida diaria

^**^AIVD: Actividades instrumentales de la vida diaria

Específicamente se encontró mayor proporción de maltrato psicológico (13.1%) seguido de negligencia (8.5%) maltrato físico (2.0%) económico (1.3%) y sexual (0.2%). En todos los tipos de maltrato las mujeres presentaron mayor proporción que los hombres con diferencias significativas (*p* <0.000). Según estratos socioeconómicos, hay un gradiente claro, a mayor estrato menor proporción de maltrato (*p* <0.000); en todos los tipos de maltrato hay mayor proporción de personas en el estrato bajo. En la zona rural se reportó mayor proporción de maltrato que la urbana, con diferencias significativas (*p* <0.000). En la Tabla 1 se muestra la distribución del tipo de maltrato según características sociodemográficas. 

En cuanto a convivientes 9.1% viven solos, 49.6% con el cónyuge, 59.7% con los hijos, 37.5% con nietos, 17.4%, con parientes y/o hermanos y 2.0%, con amigos. El promedio general de convivientes fue 3.1. De los que reportan maltrato 13.9% viven solos, casi la mitad con el cónyuge, más de la mitad con los hijos, una tercera parte con nietos, menos de una cuarta parte con hermanos u otros parientes y una pequeña proporción con amigos, con diferencias significativas en todos los convivientes. A mayor número de convivientes menor proporción de maltrato (*p* <0.000). En casi todos los tipos de maltrato hay una mayor proporción de personas maltratadas que viven con los hijos (*p* <0.000), excepto en abuso sexual que la mayor proporción corresponde a quienes viven con nietos (45.9%). Quienes viven con el cónyuge también reportan altas prevalencias de maltrato que van de 35.4% a 49.2%. Las personas que viven solas también reportan maltrato, alrededor de una quinta parte, con menores porcentajes de maltrato físico (9.8%) y mayores de abuso económico (26.6%). 

En cuanto a la funcionalidad, 78.8% son independientes en ABVD y 61.6% en AIVD. De las personas que reportaron algún tipo de maltrato 23.3% son dependientes en ABVD y 34.0% en AIVD, ambas con diferencias estadísticas significativas (*p* <0.000). Los porcentajes de maltrato son mayores en quienes tienen dependencia en AIVD excepto para el abuso sexual que es mayor en quienes tienen dependencia en ABVD (49.3%). 

Aunque todas las variables muestran diferencias significativas entre quienes reportan maltrato y quienes no, el análisis de regresión de Poisson muestra que ser mujer, la dependencia en ABVD y AIVD, vivir en zona urbana y pertenecer a estratos 1 y 2 incrementa la probabilidad de sufrir algún tipo de maltrato (*p* <0.000). Las razones de prevalencia se muestran en la Tabla 2. 

**Tabla 2:** Razones de Prevalencia del maltrato en adultos mayores, ajustadas por covariables.

Variable	Numero de maltratos	
	RP	IC 95%
**Sexo**		
Mujeres	1.13	1.13-1.14
**Region**		
Bogotá	0.69	0.68- 0.70
Pacifica	0.59	0.58-0.60
Oriental	0.51	0.51-0.52
Central	0.46	0.45-0.46
Atlántica	0.37	0.36-0.37
Orinoquia-amazonia	ref	
**Zona**		
Urbana	1.06	1.05-1.06
**Estrato**		
Bajo (1 y 2)	1.30	1.29-1.30
**Convivientes**		
Solo	0.47	0.47- 0.47
Cónyuge	0.97	0.96 - 0.97
Hijos	0.97	0.97-0.98
Nietos	0.94	0.94 - 0.95
Parientes/hermanos	0.82	0.81-0.82
**Funcionalidad **		
Dependencia en ABVD*	1.53	1.53-1.54
Dependencia en AIVD**	1.31	1.30-1.32

RP, razones de prevalencia estimadas; Modelo ajustados por región, zona y dependencia en ABVD y AIVD, convivientes: cónyuge, hijos, nietos, parientes/hermanos y amigos.

* ABVD: Actividades básicas de la vida diaria

**AIVD: Actividades instrumentales de la vida diaria

## Discusión

Este estudio revela que el hogar es un lugar de riesgo para los adultos mayores, especialmente aquellos con dependencia funcional, que viven en zonas urbanas, de estratos bajos y mujeres. 

La prevalencia de maltrato en este estudio (15.1%) es similar a lo reportado por Yon et al ^7^ quienes mediante un meta análisis que incluyo 52 estudios publicados entre 2002 y 2015 y que utilizan la misma definición del presente estudio, estiman la prevalencia global de maltrato a personas mayores en 15.7%, y aunque menciona considerables variaciones regionales, específicamente en la región de las américas la prevalencia estimada no difiere de la global. 

El tipo de maltrato más frecuente fue el psicológico y el de menor frecuencia el sexual, hallazgos similares a los reportados en la literatura ^5^^,^^7^. El estudio de Yon et al ^7^ muestra prevalencias similares a las encontradas en Colombia, de acuerdo con ellos, entre los tipos más frecuentes de maltrato están: psicológico 11.6%, abuso económico 6.8%, negligencia 4.2%, maltrato físico, 2.6% y abuso sexual 0.9%. Estas cifras difieren un poco de lo encontrado en adultos mayores colombianos, en quienes se encontró menor proporción de maltrato sexual y económico, igual proporción de maltrato físico, similar de maltrato psicológico y mayor porporción de negligencia. Si se compara con datos de Estados Unidos ^26^, es claro que la prevalencia de maltrato psicológico y de negligencia son mayores en Colombia. Tal como se menciona en la literatura, las diferencias en las prevalencias globales y de diferentes tipos de maltrato pueden deberse a problemas en el reporte o subreporte por falta de conocimiento, falta de experiencia, vergüenza, miedo a las represalias, a equivocarse, a que se incremente el maltrato o porque existe un vínculo afectivo ^26^^-^^29^. También se ha mencionado que las formas de registro y medición son insuficientes y poco desarrolladas en la región ^30^. De hecho, Acierno *et al*., encontraron que aproximadamente el 11% de los adultos mayores experimenta maltrato cada año, pero solo 1 de cada 14 casos se informa a las autoridades y se sugiere que por cada caso documentado de maltrato, 24 casos no se informan ^27^^,^^31^. Según el Primer informe intersectorial, violencias contra las personas mayores de la alcaldía Mayor de Bogotá ^32^, algunas situaciones de maltrato son evidentes sobre todo en los casos relacionados con aspectos físicos y descuido de la persona mayor, lo cual hace más fácil su identificación e intervención por parte de la sociedad y el Estado; pero otros tipos de violencia como el abuso sexual, psíquico, emocional o económico son más difíciles de detectar y no son denunciados, ya sea porque la persona mayor se ha habituado a este tipo de relación, por temor a perder la red de apoyo o por la naturalización de esta situación al interior de las familias ^32^. 

De otro lado, entre los hallazgos se destaca el hecho de que la dependencia para realizar actividades de la vida diaria básicas e instrumentales tiene las más altas razones de prevalencia en este estudio. Existe evidencia que muestra la relación entre maltrato y dependencia, especialmente, dentro del contexto familiar ^16^^,^^33^. Varios estudios constatan la asociación entre dependencia para las ABVD y maltrato ^16^^,^^29^^,^^34^. Al presentarse la dependencia se incrementa la demanda de apoyo por parte de las personas mayores, que en la mayoría de los casos es proporcionada de manera exclusiva parte de los familiares, quienes que se encuentran expuestos a situaciones de estrés, tensiones y conflictos, pues generalmente es solo una persona la que brinda el apoyo requerido. De otro lado, como las AIVD se refieren a la independencia del anciano dentro de la casa y, especialmente en la comunidad, con alto grado de complejidad para su realización ^35^, esta puede ser una de las explicaciones para que la dependencia en la AIVD esté asociada al maltrato, dado que el anciano tiene limitaciones para salir, usar transporte o mantener una red de apoyo y recurrir a alguna ayuda para salir de la situación de maltrato ^13^. La dependencia para las AIVD limita la participación social del anciano de forma independiente, restringiéndolo a contexto de su domicilio, puede además, disminuir su convivencia con otras personas, además de sus familiares, dificultando la demanda deservicios de salud y servicios especializados para denunciar cuando son sometidos a maltrato ^13^. 

Se ha sugerido que la discapacidad es tanto factor de riesgo como consecuencia del maltrato, el riesgo se atribuye a una serie de factores contextuales y sociales complejos. La mayor dependencia para el cuidado, la vulnerabilidad física, el aislamiento social y la falta de independencia económica hacen que las personas con discapacidad sean más vulnerables ^36^ En las primeras teorías sobre este tema también se intentó establecer si existía una relación entre la dependencia y el mayor riesgo de maltrato ^37^. Inicialmente, se hizo hincapié en la relación de dependencia de la víctima con la persona que la cuida o el agresor, aunque estudios de casos posteriores permitieron detectar situaciones en que los agresores dependían de la persona mayor; por lo general, hijos adultos que dependen de sus padres ancianos para el alojamiento o el sustento ^38^. En algunos de estos casos resultó evidente la existencia de una “maraña de interdependencias”, esto es, un vínculo emocional muy fuerte entre la víctima y el agresor, que muchas veces dificulta la intervención ^37^. También se ha postulado que la dependencia de los mayores puede obligar al cuidador a recordar las hostilidades pasadas, lo que resulta en maltrato al adulto mayor ^31^. 

En cuanto al estrato socioeconomico, en este estudio el reporte de maltrato es considerablemente alto en los estratos 1 y 2 (71.7%) y también es un factor predictor (RP: 1.30), sin embargo hay pocos estudios que lo refieren, un estudio de Reino Unido ^39^, aunque no considera el estrato sino la posición socieconómica, encontró tasas más altas entre los que habían estado en ocupaciones semirrutinarias y rutinarias (4.1%) en comparación con aquellos que habían sido empleadores pequeños y trabajadores por cuenta propia (0.1%), y aunque las cifras no son comparables son muy inferiores a las encontradas en este estudio. De otro lado, en cuanto al nivel de escolaridad, en Colombia, Se encontró una asociación inversamente proporcional entre nivel de escolaridad y violencia contra población adulta mayor ^14^. 

El informe Mundial sobre Violencia y Salud ^37^ traza un vínculo entre la pobreza y la violencia y describen la manera en que una vida familiar disfuncional, la carencia de dinero para los artículos esenciales y la falta de educación y de oportunidades laborales, contribuyen a que los jóvenes se dediquen al delito, el tráfico de drogas y la prostitución. En este tipo de sociedades, las personas mayores se consideran como un blanco para el maltrato y la explotación, pues su vulnerabilidad es el resultado de una situación de pobreza caracterizada por la carencia de una jubilación y de oportunidades de trabajo, la falta de higiene, la enfermedad y la mala nutrición.

En lo que respecta al sexo, el maltrato en las mujeres es más frecuente que en los hombres entre los adultos mayores ^19^^,^^20^. En todo el mundo, los hombres tienen mayores probabilidades que las mujeres de sufrir violencia en el contexto de conflictos armados y actividades delictivas, mientras que las mujeres tienen mayores probabilidades de sufrir maltrato por parte de personas cercanas, como sus cónyuges y su familia ^37^. Esta asimetría de género en el maltrato dentro del hogar parece ocurrir como resultado de la expresión de una historia de violencia, o como respuesta a un conflicto que ha estado presente en la relación a lo largo de la vida, pero que se agudiza cuando ocurre algo que altera el equilibrio, como ocurre con la jubilación ^40^. Las mujeres mayores pueden tener mas barreras para buscar ayuda y pueden ser menos conscientes del apoyo disponible ^1^. Es importante considerar que las mujeres mayores de hoy crecieron en sociedades con roles de género marcados y aprendieron a ser subordinadas ^41^. Así, la mujer mayor tolera más y está menos preparada para dejar las relaciones abusivas ^40^. La aceptabilidad de la violencia contra la mujer infligida por el compañero íntimo está generalizada, pero varía según el entorno; ademas, está generalizado en la región el acuerdo con normas que fortalecen las desigualdades de género, desaniman a las mujeres a pedir ayuda o restan importancia a la responsabilidad de terceros de intervenir en situaciones de malos tratos ^40^. 

Según datos de Medicina legal, Para el año 2018 en Colombia, en términos generales, para todas las categorías de la violencia intrafamiliar las mujeres fueron las más victimizadas; además, las personas entre los 60 y 64 años son las más perjudicadas; el riesgo aumenta si se tiene educación básica primaria y se es casado; es importante anotar que para esta entidad el término violencia intrafamiliar hace referencia a cualquier forma de maltrato, ya sea físico, psicológico o sexual, que tiene lugar entre los miembros de una familia; como todo maltrato, implica un desequilibrio de poder, y es ejercido desde el más fuerte hacia el más débil con el fin último de ejercer un control sobre la relación. Tradicionalmente, en nuestra sociedad, dentro de la estructura familiar jerárquica actualmente predominante, los dos principales ejes de desequilibrio los han constituido el género y la edad, siendo las mujeres, los niños y los ancianos las principales víctimas de la violencia dentro de la familia ^42^. 

De otro lado, actualmente se evidencia la existencia de nuevos arreglos al interior de las familias y la creciente y compleja diversidad familiar, si bien continúa predominando el tipo de familia nuclear (pareja con hijos), también se encuentran formas no comunes de organización familiar, por ejemplo: abuelos (en pareja o solos) viviendo con sus nietos, abuelos con nietos y bisnietos; hogares monoparentales simples compuestos y extensos, entre otros. La realidad que enfrentan las familias respecto al cuidado de las personas adultas mayores es complicada, los cambios generados en el interior, además el sistema centrado en la familia, provocan una gran sobrecarga para las familias, ya que son éstas quienes asumen las responsabilidades del cuidado. De igual forma, la ausencia de políticas publicas que den soporte a esta labor, hace que las personas adultas mayores dependientes sean aún más vulnerables a la probabilidad de sufrir maltrato, tanto en la familia como en las instituciones que proveen cuidados ^43^ . Bajo este contexto, la familia es el principal pilar que provee apoyo y de alguna manera cubre los riesgos sociales derivados del proceso de envejecimiento, no obstante, al interior de la familia se han presentado transformaciones, en este sentido, al no existir un soporte sólido por parte del Estado que contribuya a mejorar las condiciones de vida de las personas adultas mayores y de sus familias, esta última es objeto de una sobrecarga en términos económicos, sociales y de cuidado, que muchos de los casos derivan en maltrato. 

En este contexto familiar se ha encontrado que generalmente suelen ser los familiares directos los principales perpetradores de maltrato contra las personas adultas mayores, especialmente por los hijos/as y el cónyuge o compañero ^42^. Tambien se ha sugerido que el maltrato proviene de la dependencia del cuidador del anciano, ya sea financieramente, para el transporte o para la vivienda, no de la dependencia de la víctima sobre el abusador ^44^^,^^45^. Varios autores han sugerido un cambio de enfoque de la víctima al abusador ^44^^-^^46^ y que la interdependencia de roles podria explicar la agresión hacia los mayores ^47^, asi, el maltrato puede ser una manifestación de la dependencia de los abusadores y no de los ancianos, siendo la causa más probable un desequilibrio de poder que involucra el "déficit de poder percibido" de un niño adulto ^48^. Los miembros abusivos de la familia pueden depender de los ancianos para vivienda, transporte y apoyo financiero, y el desequilibrio de poder percibido puede ser especialmente difícil para los hijos adultos o nietos que conviven con el anciano; los miembros de la familia pueden recurrir a la agresión para tratar de obtener una ventaja de poder y superar su déficit de poder percibido. Muchos ancianos no abandonan las situaciones abusivas y a menudo niegan que el abuso esté ocurriendo, lo que sugiere que ellos también están recibiendo algo de la relación de intercambio ^44^. El Modelo de Dinámica de Cuidado Familiar sugiere que la interdependencia de roles es una explicación parcial de este fenómeno ^49^. En las familias, tanto los miembros como los ancianos dependen uno del otro para el amor, el respeto, el compañerismo y el apoyo emocional. Esta interdependencia de roles crea un ciclo de refuerzo negativo, que no permite que ninguno de los miembros salga de la relación y el maltrato continúe ^48^^,^^49^. De otro lado, de acuerdo con los reportes del Instituto de Medicina Legal ^14^ el concepto de transmisión intergeneracional de la violencia se ha usado para explicar cómo la violencia es aprendida en el contexto de socialización de la familia; es decir, la observación de la violencia en el contexto familiar puede influir en que los niños y niñas aprendan a ejercer la violencia contra otras personas ^50^. Se ha demostrado una asociación significativa entre el maltrato o abuso actual y los antecedentes familiares, lo que da lugar al fenómeno de reproducción intergeneracional de la violencia ^51^.

Finalmente, en este estudio se encontró mayor porcentaje de personas maltratadas en el grupo de menor edad, de 60 a 70 años, al igual que en lo reportado en Colombia ^14^ y en otros estudios ^1^, se ha postulado que es en este grupo de edad de ancianos más jóvenes que surgen las principales denuncias de maltrato ^4^^,^^19^. En esa franja etaria, gran parte de los ancianos es activa física e intelectualmente, tiene más autonomía, independencia y condiciones para buscar ayuda. En cambio, la literatura científica relata que las mayores víctimas son los ancianos mayores, debido a las limitaciones funcionales y cognitivas ^5^^,^^31^

Una de las mayores fortalezas del estudio es su carácter poblacional que hace que los resultados se pueden generalizar a todo el país. En nuestro conocimiento, es el primer estudio colombiano que muestra la prevalencia de maltrato de forma general y especifica por tipos de maltrato y analiza sus relaciones con la region, la zona geografica, el estrato socioeconomico, los arreglos familiares y la dependencia funcional en adultos mayores. 

Entre las limitaciones del estudio se encuentra que los datos fueron registrados por auto-reporte con una escala de que no mide ni la intencionalidad ni la severidad del maltrato, lo cual impide estimar de manera más precisa las diferencias en la experiencia y perpetuación. Tampoco se identifico de manera precisa la fuente especifica del maltrato. Finalmente, éste es un estudio transversal y cuantitativo sobre el maltrato. Futuros estudios sobre el tema deberán incluir un abordaje cualitativo, así como metodologías que puedan establecer relaciones de causalidad.

## Conclusiones

El maltrato en personas mayores debe ser prioritaria para las agendas de políticas públicas y de salud en el país. Este estudio revela que el hogar es un lugar de riesgo para los adultos mayores, especialmente aquellos con dependencia funcional, de estratos bajos y mujeres. Desde una perspectiva social, la consecuencia más grave de la violencia será el aislamiento, la disminución de autoestima y la presencia de sentimientos de inseguridad, que favorecerán la creación de estereotipos negativos de la vejez. Desde la perspectiva de salud pública, el maltrato hacia las personas mayores puede tener consecuencias graves; el adulto mayor aislado y sin apoyo social es más vulnerable y tendrá mayor morbilidad y discapacidad. Estos resultados deberán animar el debate sobre los patrones de relación existentes en las familias latinoamericanas y sobre el rol de la mujer en ellas, y pensar en los medios necesarios para transformar las dinámicas familiares violentas en hogares con personas mayores.
